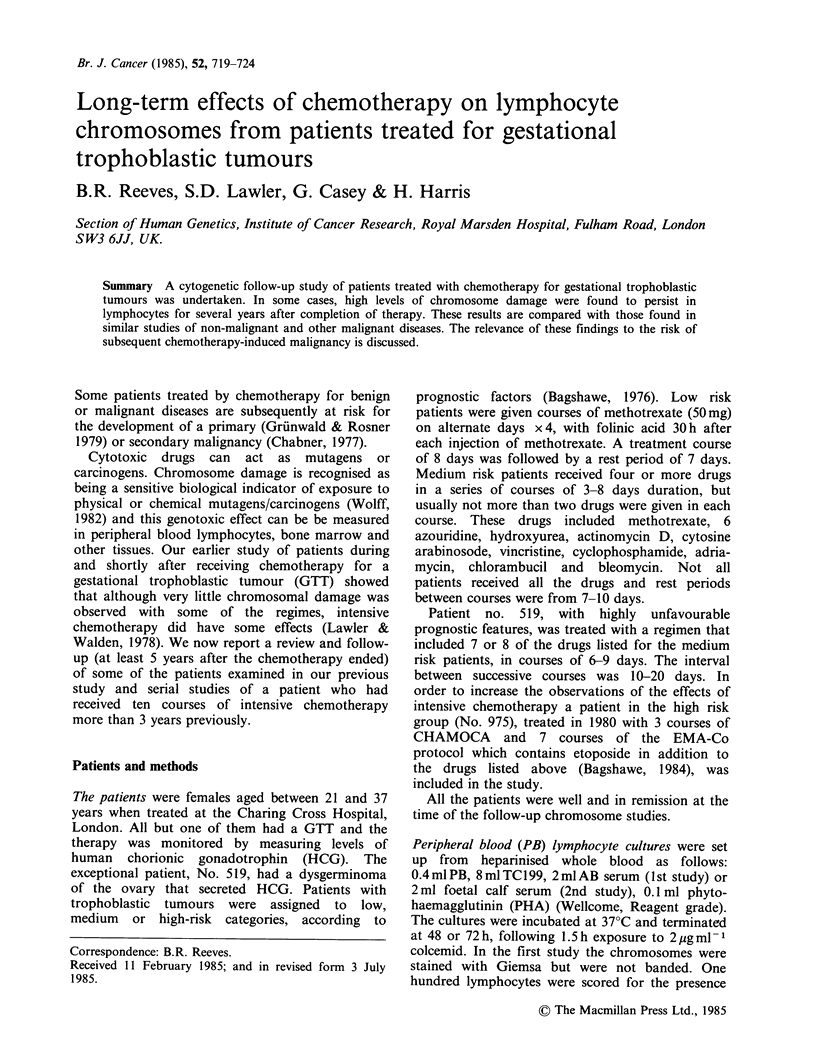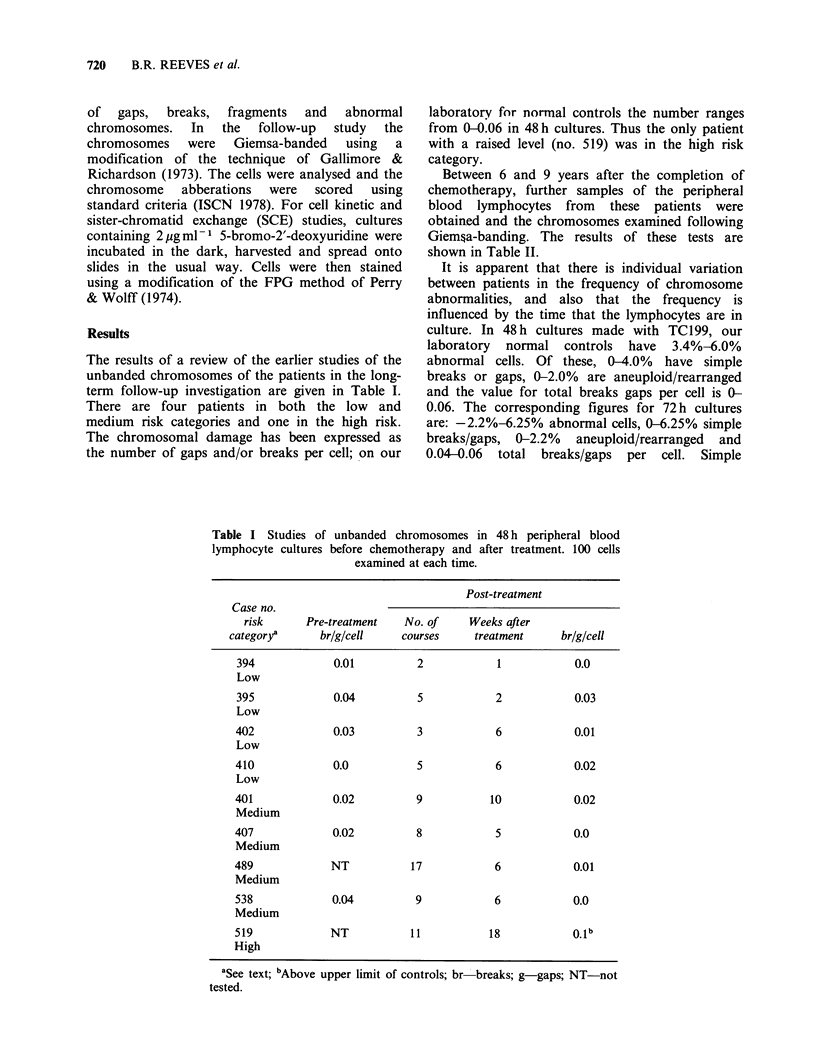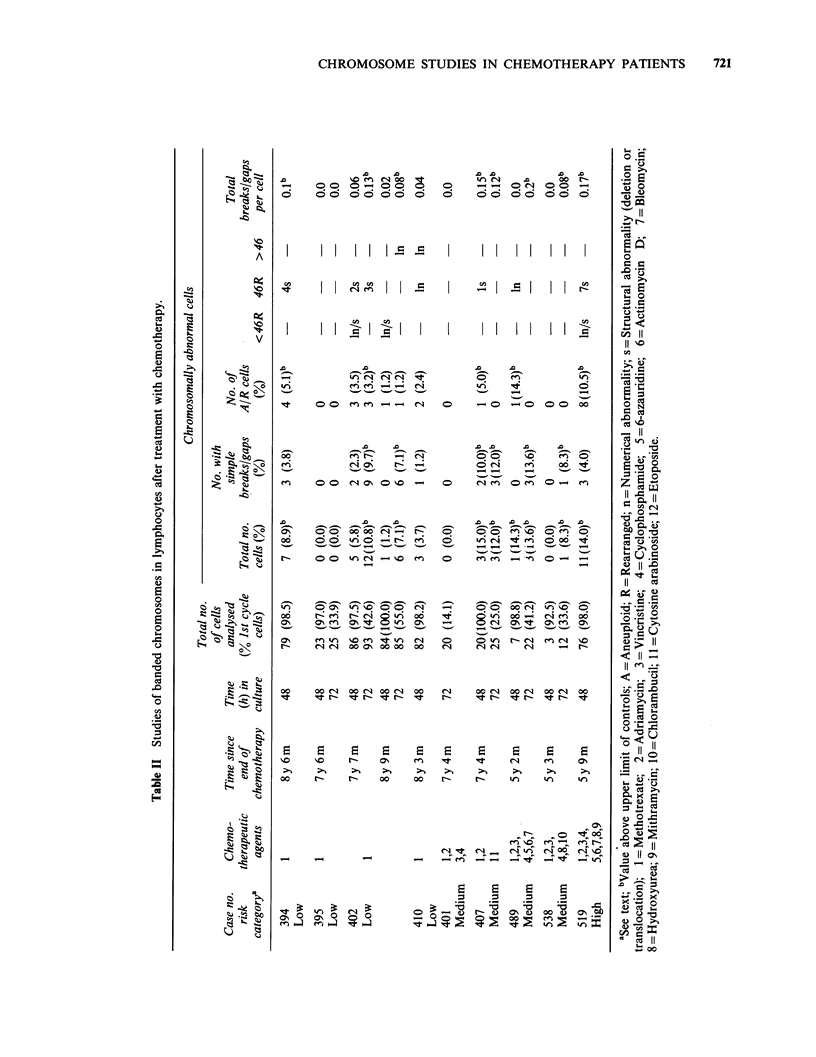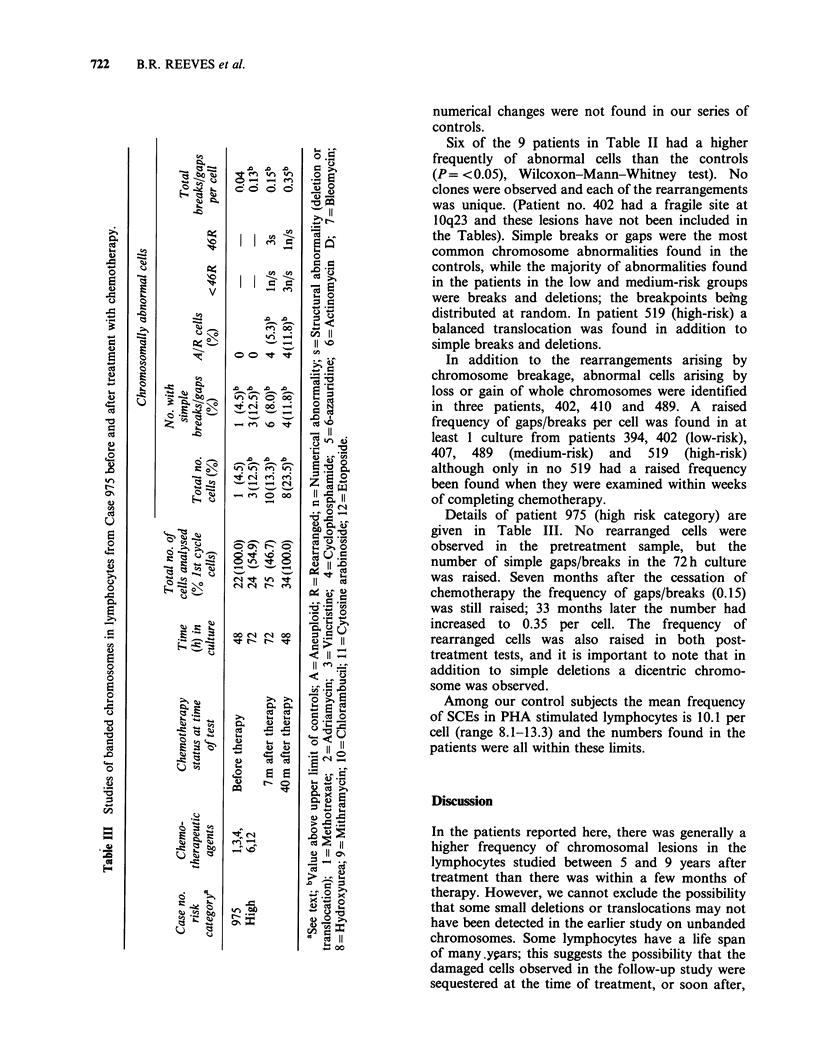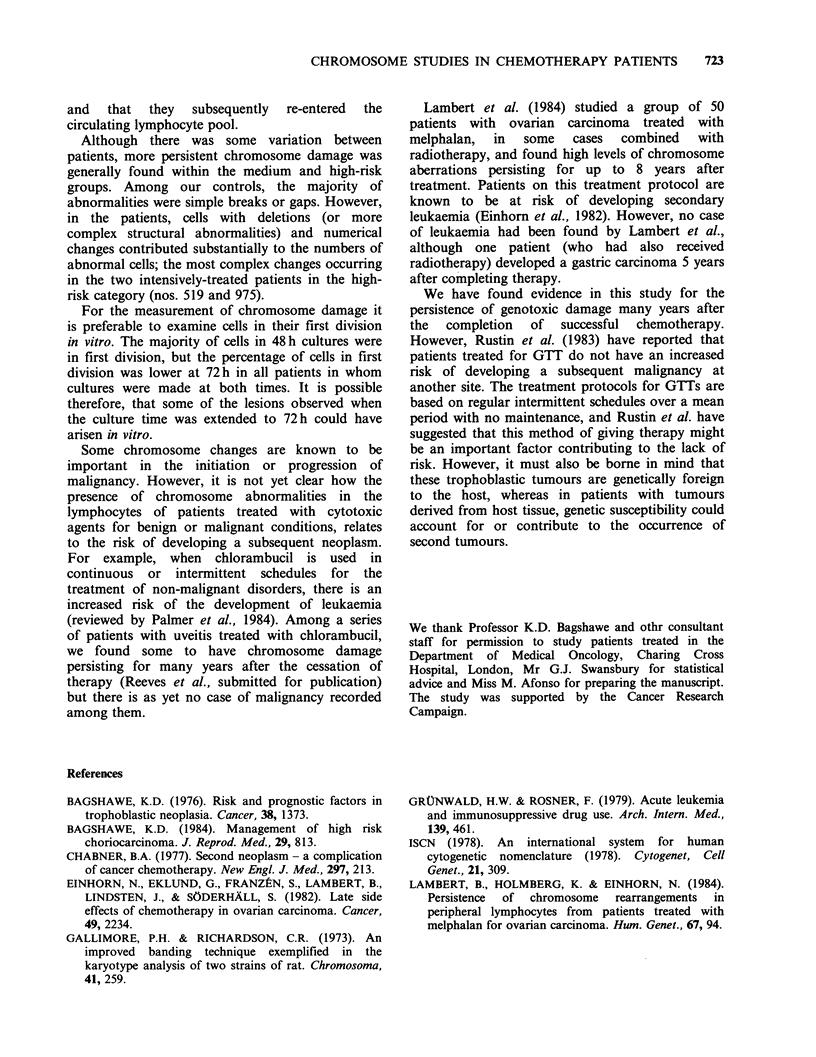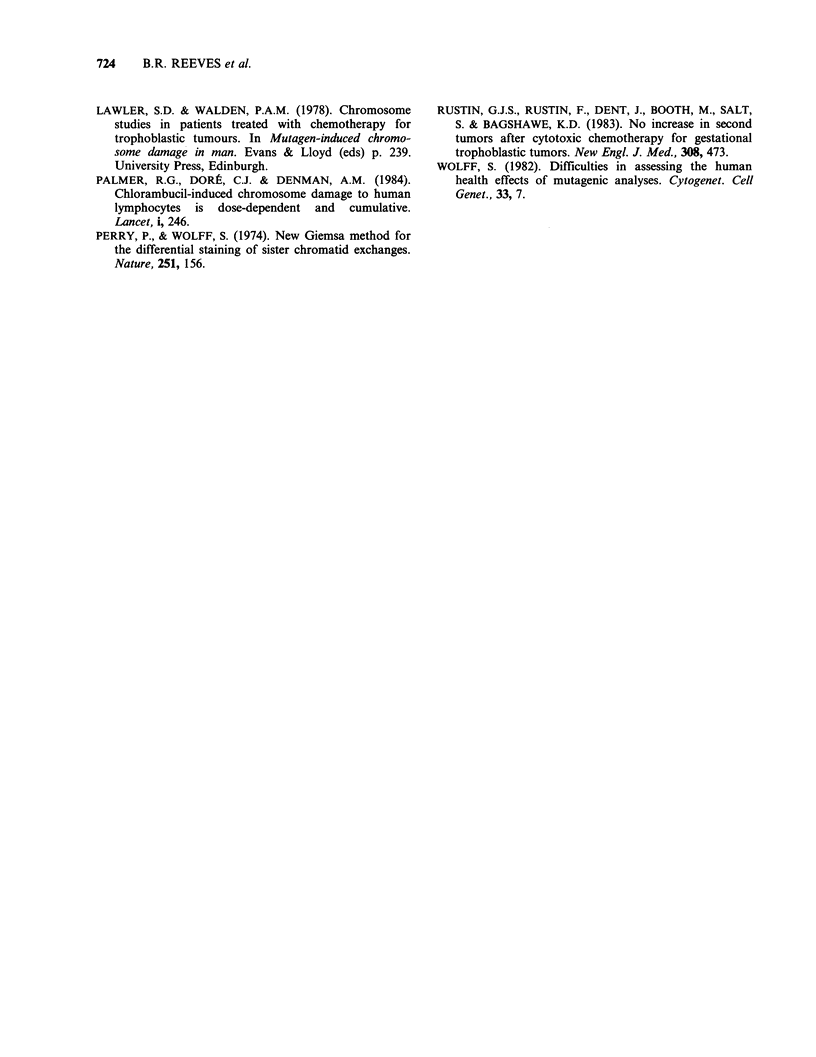# Long-term effects of chemotherapy on lymphocyte chromosomes from patients treated for gestational trophoblastic tumours.

**DOI:** 10.1038/bjc.1985.249

**Published:** 1985-11

**Authors:** B. R. Reeves, S. D. Lawler, G. Casey, H. Harris

## Abstract

A cytogenetic follow-up study of patients treated with chemotherapy for gestational trophoblastic tumours was undertaken. In some cases, high levels of chromosome damage were found to persist in lymphocytes for several years after completion of therapy. These results are compared with those found in similar studies of non-malignant and other malignant diseases. The relevance of these findings to the risk of subsequent chemotherapy-induced malignancy is discussed.


*Br. J. Cancer* (1985), **52**, 719–724

### Long-term effects of chemotherapy on lymphocyte chromosomes from patients treated for gestational trophoblastic tumours

B.R. Reeves, S.D. Lawler, G. Casey & H. Harris

*Section of Human Genetics, Institute of Cancer Research, Royal Marsden Hospital, Fulham Road, London SW3 6JJ, UK.*

**Summary** A cytogenetic follow-up study of patients treated with chemotherapy for gestational trophoblastic tumours was undertaken. In some cases, high levels of chromosome damage were found to persist in lymphocytes for several years after completion of therapy. These results are compared with those found in similar studies of non-malignant and other malignant diseases. The relevance of these findings to the risk of subsequent chemotherapy-induced malignancy is discussed.

Some patients treated by chemotherapy for benign or malignant diseases are subsequently at risk for the development of a primary (Grünwald & Rosner 1979) or secondary malignancy (Chabner, 1977).

Cytotoxic drugs can act as mutagens or carcinogens. Chromosome damage is recognised as being a sensitive biological indicator of exposure to physical or chemical mutagens/carcinogens (Wolff, 1982) and this genotoxic effect can be be measured in peripheral blood lymphocytes, bone marrow and other tissues. Our earlier study of patients during and shortly after receiving chemotherapy for a gestational trophoblastic tumour (GTT) showed that although very little chromosomal damage was observed with some of the regimes, intensive chemotherapy did have some effects (Lawler & Walden, 1978). We now report a review and follow-up (at least 5 years after the chemotherapy ended) of some of the patients examined in our previous study and serial studies of a patient who had received ten courses of intensive chemotherapy more than 3 years previously.

### Patients and methods

*The patients* were females aged between 21 and 37 years when treated at the Charing Cross Hospital, London. All but one of them had a GTT and the therapy was monitored by measuring levels of human chorionic gonadotrophin (HCG). The exceptional patient, No. 519, had a dysgerminoma of the ovary that secreted HCG. Patients with trophoblastic tumours were assigned to low, medium or high-risk categories, according to prognostic factors (Bagshawe, 1976). Low risk patients were given courses of methotrexate (50 mg) on alternate days ×4, with folinic acid 30 h after each injection of methotrexate. A treatment course of 8 days was followed by a rest period of 7 days. Medium risk patients received four or more drugs in a series of courses of 3–8 days duration, but usually not more than two drugs were given in each course. These drugs included methotrexate, 6 azouridine, hydroxyurea, actinomycin D, cytosine arabinosode, vincristine, cyclophosphamide, adriamycin, chlorambucil and bleomycin. Not all patients received all the drugs and rest periods between courses were from 7–10 days.

Patient no. 519, with highly unfavourable prognostic features, was treated with a regimen that included 7 or 8 of the drugs listed for the medium risk patients, in courses of 6–9 days. The interval between successive courses was 10–20 days. In order to increase the observations of the effects of intensive chemotherapy a patient in the high risk group (No. 975), treated in 1980 with 3 courses of CHAMOCA and 7 courses of the EMA-Co protocol which contains etoposide in addition to the drugs listed above (Bagshawe, 1984), was included in the study.

All the patients were well and in remission at the time of the follow-up chromosome studies.

*Peripheral blood (PB) lymphocyte cultures* were set up from heparinised whole blood as follows: 0.4 ml PB, 8 ml TC199, 2 ml AB serum (1st study) or 2 ml foetal calf serum (2nd study), 0.1 ml phytohaemagglutinin (PHA) (Wellcome, Reagent grade). The cultures were incubated at 37°C and terminated at 48 or 72 h, following 1.5 h exposure to $2\,\mu g\,ml^{-1}$ colcemid. In the first study the chromosomes were stained with Giemsa but were not banded. One hundred lymphocytes were scored for the presence

Correspondence: B.R. Reeves.
Received 11 February 1985; and in revised form 3 July 1985.

© The Macmillan Press Ltd., 1985

of gaps, breaks, fragments and abnormal chromosomes. In the follow-up study the chromosomes were Giemsa-banded using a modification of the technique of Gallimore & Richardson (1973). The cells were analysed and the chromosome abberations were scored using standard criteria (ISCN 1978). For cell kinetic and sister-chromatid exchange (SCE) studies, cultures containing $2 \mu g ml^{-1}$ 5-bromo-2′-deoxyuridine were incubated in the dark, harvested and spread onto slides in the usual way. Cells were then stained using a modification of the FPG method of Perry & Wolff (1974).

### Results

The results of a review of the earlier studies of the unbanded chromosomes of the patients in the long-term follow-up investigation are given in Table I. There are four patients in both the low and medium risk categories and one in the high risk. The chromosomal damage has been expressed as the number of gaps and/or breaks per cell; on our laboratory for normal controls the number ranges from 0–0.06 in 48 h cultures. Thus the only patient with a raised level (no. 519) was in the high risk category.

Between 6 and 9 years after the completion of chemotherapy, further samples of the peripheral blood lymphocytes from these patients were obtained and the chromosomes examined following Giemsa-banding. The results of these tests are shown in Table II.

It is apparent that there is individual variation between patients in the frequency of chromosome abnormalities, and also that the frequency is influenced by the time that the lymphocytes are in culture. In 48 h cultures made with TC199, our laboratory normal controls have 3.4%–6.0% abnormal cells. Of these, 0–4.0% have simple breaks or gaps, 0–2.0% are aneuploid/rearranged and the value for total breaks gaps per cell is 0–0.06. The corresponding figures for 72 h cultures are: −2.2%–6.25% abnormal cells, 0–6.25% simple breaks/gaps, 0–2.2% aneuploid/rearranged and 0.04–0.06 total breaks/gaps per cell. Simple

**Table I** Studies of unbanded chromosomes in 48 h peripheral blood lymphocyte cultures before chemotherapy and after treatment. 100 cells examined at each time.

| Case no. risk category[a] | Pre-treatment br/g/cell | Post-treatment No. of courses | Weeks after treatment | br/g/cell |
|---|---|---|---|---|
| 394 Low | 0.01 | 2 | 1 | 0.0 |
| 395 Low | 0.04 | 5 | 2 | 0.03 |
| 402 Low | 0.03 | 3 | 6 | 0.01 |
| 410 Low | 0.0 | 5 | 6 | 0.02 |
| 401 Medium | 0.02 | 9 | 10 | 0.02 |
| 407 Medium | 0.02 | 8 | 5 | 0.0 |
| 489 Medium | NT | 17 | 6 | 0.01 |
| 538 Medium | 0.04 | 9 | 6 | 0.0 |
| 519 High | NT | 11 | 18 | 0.1[b] |

[a]See text; [b]Above upper limit of controls; br—breaks; g—gaps; NT—not tested.

numerical changes were not found in our series of controls.

Six of the 9 patients in Table II had a higher frequently of abnormal cells than the controls ($P = <0.05$), Wilcoxon–Mann–Whitney test). No clones were observed and each of the rearrangements was unique. (Patient no. 402 had a fragile site at 10q23 and these lesions have not been included in the Tables). Simple breaks or gaps were the most common chromosome abnormalities found in the controls, while the majority of abnormalities found in the patients in the low and medium-risk groups were breaks and deletions; the breakpoints being distributed at random. In patient 519 (high-risk) a balanced translocation was found in addition to simple breaks and deletions.

In addition to the rearrangements arising by chromosome breakage, abnormal cells arising by loss or gain of whole chromosomes were identified in three patients, 402, 410 and 489. A raised frequency of gaps/breaks per cell was found in at least 1 culture from patients 394, 402 (low-risk), 407, 489 (medium-risk) and 519 (high-risk) although only in no 519 had a raised frequency been found when they were examined within weeks of completing chemotherapy.

Details of patient 975 (high risk category) are given in Table III. No rearranged cells were observed in the pretreatment sample, but the number of simple gaps/breaks in the 72 h culture was raised. Seven months after the cessation of chemotherapy the frequency of gaps/breaks (0.15) was still raised; 33 months later the number had increased to 0.35 per cell. The frequency of rearranged cells was also raised in both post-treatment tests, and it is important to note that in addition to simple deletions a dicentric chromosome was observed.

Among our control subjects the mean frequency of SCEs in PHA stimulated lymphocytes is 10.1 per cell (range 8.1–13.3) and the numbers found in the patients were all within these limits.

### Discussion

In the patients reported here, there was generally a higher frequency of chromosomal lesions in the lymphocytes studied between 5 and 9 years after treatment than there was within a few months of therapy. However, we cannot exclude the possibility that some small deletions or translocations may not have been detected in the earlier study on unbanded chromosomes. Some lymphocytes have a life span of many years; this suggests the possibility that the damaged cells observed in the follow-up study were sequestered at the time of treatment, or soon after, and that they subsequently re-entered the circulating lymphocyte pool.

Although there was some variation between patients, more persistent chromosome damage was generally found within the medium and high-risk groups. Among our controls, the majority of abnormalities were simple breaks or gaps. However, in the patients, cells with deletions (or more complex structural abnormalities) and numerical changes contributed substantially to the numbers of abnormal cells; the most complex changes occurring in the two intensively-treated patients in the high-risk category (nos. 519 and 975).

For the measurement of chromosome damage it is preferable to examine cells in their first division *in vitro*. The majority of cells in 48 h cultures were in first division, but the percentage of cells in first division was lower at 72 h in all patients in whom cultures were made at both times. It is possible therefore, that some of the lesions observed when the culture time was extended to 72 h could have arisen *in vitro*.

Some chromosome changes are known to be important in the initiation or progression of malignancy. However, it is not yet clear how the presence of chromosome abnormalities in the lymphocytes of patients treated with cytotoxic agents for benign or malignant conditions, relates to the risk of developing a subsequent neoplasm. For example, when chlorambucil is used in continuous or intermittent schedules for the treatment of non-malignant disorders, there is an increased risk of the development of leukaemia (reviewed by Palmer *et al.*, 1984). Among a series of patients with uveitis treated with chlorambucil, we found some to have chromosome damage persisting for many years after the cessation of therapy (Reeves *et al.*, submitted for publication) but there is as yet no case of malignancy recorded among them.

Lambert *et al.* (1984) studied a group of 50 patients with ovarian carcinoma treated with melphalan, in some cases combined with radiotherapy, and found high levels of chromosome aberrations persisting for up to 8 years after treatment. Patients on this treatment protocol are known to be at risk of developing secondary leukaemia (Einhorn *et al.*, 1982). However, no case of leukaemia had been found by Lambert *et al.*, although one patient (who had also received radiotherapy) developed a gastric carcinoma 5 years after completing therapy.

We have found evidence in this study for the persistence of genotoxic damage many years after the completion of successful chemotherapy. However, Rustin *et al.* (1983) have reported that patients treated for GTT do not have an increased risk of developing a subsequent malignancy at another site. The treatment protocols for GTTs are based on regular intermittent schedules over a mean period with no maintenance, and Rustin *et al.* have suggested that this method of giving therapy might be an important factor contributing to the lack of risk. However, it must also be borne in mind that these trophoblastic tumours are genetically foreign to the host, whereas in patients with tumours derived from host tissue, genetic susceptibility could account for or contribute to the occurrence of second tumours.

We thank Professor K.D. Bagshawe and othr consultant staff for permission to study patients treated in the Department of Medical Oncology, Charing Cross Hospital, London, Mr G.J. Swansbury for statistical advice and Miss M. Afonso for preparing the manuscript. The study was supported by the Cancer Research Campaign.

### References

BAGSHAWE, K.D. (1976). Risk and prognostic factors in trophoblastic neoplasia. *Cancer*, **38**, 1373.

BAGSHAWE, K.D. (1984). Management of high risk choriocarcinoma. *J. Reprod. Med.*, **29**, 813.

CHABNER, B.A. (1977). Second neoplasm – a complication of cancer chemotherapy. *New Engl. J. Med.*, **297**, 213.

EINHORN, N., EKLUND, G., FRANZÉN, S., LAMBERT, B., LINDSTEN, J., & SÖDERHÄLL, S. (1982). Late side effects of chemotherapy in ovarian carcinoma. *Cancer*, **49**, 2234.

GALLIMORE, P.H. & RICHARDSON, C.R. (1973). An improved banding technique exemplified in the karyotype analysis of two strains of rat. *Chromosoma*, **41**, 259.

GRÜNWALD, H.W. & ROSNER, F. (1979). Acute leukemia and immunosuppressive drug use. *Arch. Intern. Med.*, **139**, 461.

ISCN (1978). An international system for human cytogenetic nomenclature (1978). *Cytogenet, Cell Genet.*, **21**, 309.

LAMBERT, B., HOLMBERG, K. & EINHORN, N. (1984). Persistence of chromosome rearrangements in peripheral lymphocytes from patients treated with melphalan for ovarian carcinoma. *Hum. Genet.*, **67**, 94.

LAWLER, S.D. & WALDEN, P.A.M. (1978). Chromosome studies in patients treated with chemotherapy for trophoblastic tumours. In *Mutagen-induced chromosome damage in man*. Evans & Lloyd (eds) p. 239. University Press, Edinburgh.

PALMER, R.G., DORÉ, C.J. & DENMAN, A.M. (1984). Chlorambucil-induced chromosome damage to human lymphocytes is dose-dependent and cumulative. *Lancet*, **i**, 246.

PERRY, P., & WOLFF, S. (1974). New Giemsa method for the differential staining of sister chromatid exchanges. *Nature*, **251**, 156.

RUSTIN, G.J.S., RUSTIN, F., DENT, J., BOOTH, M., SALT, S. & BAGSHAWE, K.D. (1983). No increase in second tumors after cytotoxic chemotherapy for gestational trophoblastic tumors. *New Engl. J. Med.*, **308**, 473.

WOLFF, S. (1982). Difficulties in assessing the human health effects of mutagenic analyses. *Cytogenet. Cell Genet.*, **33**, 7.